# A RECURRENT MUTATION IN TSHB GENE UNDERLYING CENTRAL CONGENITAL HYPOTHYROIDISM UNDETECTABLE IN NEONATAL SCREENING

**DOI:** 10.1590/1984-0462/;2019;37;4;00017

**Published:** 2019-06-03

**Authors:** Maria de Fátima Borges, Horacio Mario Domené, Paula Alejandra Scaglia, Beatriz Hallal Jorge Lara, Heloísa Marcelina da Cunha Palhares, Andréia Vasconcelos Aguiar Santos, Amanda Lacerda Ferreira Gonçalves, Marília Matos Oliveira, Alessandra Bernadete Trovó de Marqui

**Affiliations:** a Universidade Federal do Triângulo Mineiro, Uberaba, MG, Brazil.; b Hospital de Niños Ricardo Gutiérrez, Buenos Aires, Argentina.

**Keywords:** Congenital hypothyroidism, Thyrotropin, beta subunit, Mutation, Neonatal screening, Hipotireoidismo congênito, Tireotropina subunidade beta, Mutação, Triagem neonatal

## Abstract

**Objective:**

To describe the case of a patient with central congenital hypothyroidism (CCH) due to a recurrent mutation in the TSHB gene, as well as to conduct a genetic study of his family.

**Case description:**

It is presented a case report of a 5-month-old boy with a delayed diagnosis of isolated CCH in whom the molecular analysis was performed 12 years later and detected a recurrent mutation (c.373delT) in TSHB gene. The parents and sister were carriers of the mutant allele.

**Comments:**

The c.373delT mutation has previously been reported in patients from Brazil, Germany, Belgium, United States, Switzerland, Argentina, France, Portugal, United Kingdom and Ireland. In summary, our case and other ones reported in the literature support the theory that this mutation may be a common cause of isolated TSH deficiency. Isolated TSH deficiency is not detected by routine TSH-based neonatal screening, representing a clinical challenge. Therefore, when possible, molecular genetic study is indicated. Identification of affected and carriers allows the diagnosis, treatment and adequate genetic counseling.

## RESUMO

**Objetivo:**

Descrever o caso de um paciente com hipotireoidismo congênito central (HCC) por conta de uma mutação recorrente no gene *TSHB*, bem como realizar um estudo genético de sua família.

**Descrição do caso:**

Relato de caso de um menino de 5 meses de idade com diagnóstico tardio de HCC isolado, em quem a análise molecular foi realizada 12 anos depois e detectou uma mutação recorrente (c.373delT) no gene *TSHB*. Os pais e a irmã eram portadores do alelo mutante.

**Comentários:**

A mutação c.373delT já foi relatada em pacientes do Brasil, da Alemanha, da Bélgica, dos Estados Uinidos, da Suíça, da Argentina, da França, de Portugal, do Reino Unido e da Irlanda. Em resumo, nosso caso e outros relatados na literatura reforçam a teoria de que essa mutação pode ser uma causa comum de deficiência isolada de TSH. A deficiência isolada de TSH não é detectada na triagem neonatal com base na dosagem de TSH, representando um desafio clínico. Portanto, quando possível, o estudo genético molecular é indicado. A identificação dos afetados e dos portadores permite o diagnóstico, o tratamento e o aconselhamento genético adequado.

## INTRODUCTION

Central congenital hypothyroidism (CCH; OMIM#275100) is a rare disorder in which inadequate thyroid hormone biosynthesis occurs due to defective stimulation of a normal thyroid gland by thyroid stimulating hormone (TSH). Most patients with CCH have low-free thyroxine levels and inappropriately low or normal TSH levels, although a few have slightly elevated TSH levels. As routine, neonatal screening for congenital hypothyroidism in most western countries is based on the detection of elevated levels of TSH, and newborns with CCH may not be identified.^1^

CCH may be isolated or occur as a component of combined pituitary hormone deficiencies (majority of cases).^1^ A recent review described known genetic causes of isolated central hypothyroidism and combined pituitary hormone deficits associated with TSH deficiency.^1^ Therefore, CCH can be caused by mutations in known transcription factors such as POU1F1, PROP1, HESX1, LHX3, LHX4, SOX3 and OTX2, that are implicated in pituitary development and differentiation.^2^ Isolated central hypothyroidism is a rare entity, with an estimated incidence of 1:65,000, and may occur as a result of defects in genes controlling the TSH biosynthetic pathway, comprising mutations in TSHB, TRHR and IGSF1 genes.^1^ ^,^ ^3^

CCH evades diagnosis in TSH based congenital hypothyroidism screening programs in most countries in the world. Accordingly, genetic diagnosis, enabling ascertainment of affected relatives in families, is critical for prompt diagnosis and treatment of the disorder.

The objective of the present study was to describe a patient with CCH due to a recurrent mutation in the TSHB gene, as well as to conduct a genetic study of his family.

## CASE REPORT

The study was approved by the Ethics Committee of Universidade Federal do Triângulo Mineiro (UFTM) (CAAE: 84250518.0.0000.5154). Written informed consent was obtained from all the participants.

A white male patient born in Uberaba, Minas Gerais, Brazil, who was the son of consanguineous parents (first cousins), was born post-term (43 weeks) by cesarean section, without neonatal complications, weighing 4,080 g and measuring 53 cm. He had an updated vaccination card and a normal newborn screening test (TSH=7 µUI/L; reference value ENT#091;RVENT#093;≤10 µg/dL; T4 total=11 µg/dL; RV=10-18 µg/dL).

The patient was admitted to the Pediatric Emergency Department of the Hospital das Clínicas of the UFTM at 5 months and 3 days of age and exhibited decreased appetite, crying, bloating, and drowsiness lasting for one day. The mother also reported constipation since he was 20 days old, difficulty sucking, generalized hypotonia, and changes in neuro-psychomotor development, including difficulty in steadying and moving his head.

On physical examination, the patient exhibited a normal general condition with pale skin, no fever, dry skin and hair, an ogival palate, a saddle nose, macroglossia, subcutaneous infiltration, unchanged lung and heart auscultation, and topical testicles. The patient’s weight was -2.1 standard deviations and his length was -3.6 standard deviations of the age-appropriate value. Laboratory tests confirmed the diagnosis of central hypothyroidism (TSH=1.6 mUI/mL; RV=0.38-4.50 has mUI/Ml and free T4=0.1 ng/dL; RV=0.8-2.3 ng/dL). The patient also had a low total T3 level (40 ng/dL; RV=53-205 ng/dL) and negative thyroid antibodies. The glucose, cortisol, prolactin, and growth hormone (GH) levels were within the normal range (glucose=55 mg/dL; cortisol=12.1 µg/dL; RV=5-25 µg/dL; prolactin=22.4 ng/mL; RV=5-18.5 ng/mL; GH=0.9 ng/mL; RV≥5 ng/mL). A thyroid ultrasound examination showed a topical and hypoplastic thyroid, and head computed tomography results were normal.

Levothyroxine therapy was initiated at a dose of 10 µg/kg/day with subsequent dose adjustments during follow-up. The patient progressed with complete resolution of signs and symptoms of central hypothyroidism, but with hyperactivity and cognitive impairment. He had normal height and pubertal development. Currently, at 22 years of age, he has reached the final height of 179 cm, exceeding the target height (172 cm), weight of 116 kg, body mass index (BMI) of 36.2 kg/m^2^, abdominal circumference of 118 cm and, therefore, developed metabolic syndrome.

**Figure 1:**
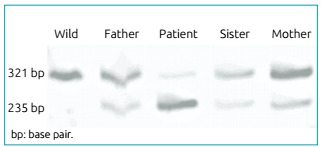
Molecular analysis of genomic deoxyribonucleic acid identified a homozygous one-base pair deletion in exon 3 of the TSHB gene that creates a new restriction site for SnaBI. The wild-type allele remained undigested (321 bp), whereas the mutant allele was cut into two fragments of 235 (as shown in the figure) and 86 bp (not shown). Our patient is homozygous for this mutation. Both parents and his sister are heterozygous.

Molecular analysis was performed in collaboration with Argentine researchers (HMD and PAS), Buenos Aires, and conducted 12 years later the clinical suspicion. Deoxyribonucleic acid (DNA) extraction from peripheral blood leucocytes, polymerase chain reaction (PCR) and restriction fragment length polymorphism (RFLP) analysis were done according to methods described elsewhere.^4^ The recurrent mutation c.373delT, identified in the patient, introduces a SnaBI restriction site in exon 3 of TSHB gene. The exon 3 was amplified by PCR from genomic DNA (amplicon size, 321 bp) and digested with SnaBI. Products were resolved by polyacrylamide gel electrophoresis, showing two digested fragments of 235 and 86 bp (not shown) for the mutant allele, while the wild type allele remains undigested. The patient was homozygous for the mutation. A molecular study was first performed on the patient, and then, after confirming the mutation, the parents and only sister were included. Peripheral blood was also collected from his parents and only sister, which resulted as heterozygous carriers of the mutant allele and had normal concentrations of TSH and free T4, displayed three fragments of 321, 235 and 86 bp (Figure 1).

## DISCUSSION

Isolated CCH is a rare variant of congenital hypothyroidism, the majority of cases is associated with mutations in the TSHB gene (OMIM#188540), and the inheritance is autosomal recessive.^1^

The TSHB gene, located on the short arm of chromosome 1 (1p13.2), has three exons, two of which encode a 138 aminoacid protein.^5^ ^,^ ^6^ Nine different TSHB gene mutations have been reported, all with clinical manifestations. A detailed review of all previously reported TSHB gene mutations is presented by Pappa et al.^7^

In the present study, a molecular analysis performed when the patient was 12 years old showed a recurrent mutation in the TSHB gene, indicating its homozygous character. The parents (first cousins) and the sister were heterozygous for the c.373delT mutation. Molecular analysis is important because it helps to extend genetic counseling to other family members.

The most frequently reported TSHB mutation is a single nucleotide deletion (c.373delT), resulting in a frameshift that leads to a cysteine 125 to valine change (p.C125V). The nomenclature for this mutation has been updated to p.C125Vfs*10 (NM_000549.4:c.373delT) and follows the most recent HUGO Gene Nomenclature Committee (HGNC) guidelines to include the 20 aminoacid signal peptide of TSHB gene, such that the annotation may differ from that one cited in the originally published articles. This mutation, located on exon 3 of the TSHB gene, was first identified in 1996^8^ and was originally described as c.313delT (protein change: C105Vfs114X). The authors performed functional studies and showed that it is the cysteine to valine aminoacid change at codon 125, rather than subsequent deletion of 13 carboxyterminal residues of TSHB, which impairs the function of mutant TSH.^8^ Our study is the second one to identify this mutation in Brazilian patients with CCH.

The c.373delT mutation has previously been reported in patients from Germany,^9^ ^,^ ^10^ ^,^ ^11^ ^,^ ^12^ ^,^ ^13^ United States^14^ ^,^ ^15^ ^,^ ^16^ (Table 1), Brazil,^8^ Belgium,^17^ Switzerland,^18^ ^,^ ^19^ Portugal,^19^ France,^19^ ^,^ ^20^ Argentina,^4^ ^,^ ^18^ ^,^ ^21^ United Kingdom and Ireland^22^ (Table 2). The occurrence of the same mutation in families and/or patients of different ethnic origin suggests that it may be prevalent in the population.

**Table 1:** Studies conducted in Germany and the United States that describe affected patients and carriers of the c.373 delT mutation of the TSHB gene, recurrent in patients with central congenital hypothyroidism.

Author	Country	Subjects
Doeker et al.^9^	Germany	5-month-old infant of nonconsanguineous parents proposita - homozygous for the mutation unaffected parents, the paternal grandmother, and the maternal grandfather - heterozygous
Biebermann et al.^10^	Germany	first child of apparently unrelated parents
Brumm et al.^11^	Germany	three nonconsanguineous families affected: family A (two patients), family B (one patient, published previously) and family C (two patients) parents - heterozygous carriers
Partsch et al.^12^	Germany	2-year-old girl of nonconsanguineous parents
Grünert et al.^13^	Germany	a female infant - homozygous for the mutation and nonconsanguineous Caucasian parents (heterozygous)
McDermott et al.^14^	United States	two adult siblings
Felner et al.^15^	United States	two sisters of Scottish-Irish ancestry
Morales et al.^16^	United States	a compound heterozygous patient - mutations at codons 57 and 125

**Table 2:** Studies conducted in other countries that describe affected patients and carriers of the c.373 delT mutation of the TSHB gene, recurrent in patients with central congenital hypothyroidism.

Author	Country	Subjects
Medeiros-Neto et al.^8^	Brazil	Two related families (family A: six children; family B: two children) with Consanguineous parents Affected members: 4 (family A: 3; family B: 1) Carriers of the mutation: 5 (family A: 2; family B: 3)
Heinrichs et al.^17^	Belgium	A 7 years old girl
Deladoëy et al.^18^	Switzerland and Argentina	Three unrelated Argentinean families Two unrelated Swiss families
Karges et al.^19^	Four european countries	One infant - compound heterozygoty for 145C→T (Q49X) and c.373delT (C125Vfs134X) (France) Five patients - homozygous mutation c.373delT (Switzerland: 1, Germany: 2 and Portugal: 2)
Ramos et al.^20^	France	One family: three affected siblings
Domené et al.^4^	Argentina	Eight affected children (three boys and five girls): homozygous for the mutation from seven unrelated families (nine parents - carriers)
Baquedano et al.^21^	Argentina	One boy: compound heterozygoty for c.313delT and c.323G>A (C88Y) Unaffected father and mother (nonconsanguineous parents): heterozygous carriers of c.313delT and C88Y mutant alleles, respectively
Nicholas et al.^22^	United Kingdom (UK) and Ireland	Four cases: three unrelated families Family 1: two siblings - homozygous for c.373delT mutation Family 2: affected child - compound heterozygous for c.373delT and c.1-4389_417*195delinsCTCA Family 3: proband - compound heterozygous for c.373delT and c.2T>C, p.Met1?

F: family.

A previous study suggested genetic founder effects in CCH caused by this mutation.^11^ Another recent study, performing haplotype analysis, to investigate a founder effect, was undertaken in cases with identical mutations (c.373delT).^22^ Both studies supported the notion of a founder effect for c373delT mutation of the TSHB gene.^11^ ^,^ ^22^ According to Deladoëy et al., the c.373delT mutation was the most frequent alteration causing CCH (13 of 22; 59%) and occurred mainly in unrelated and non-consanguineous families (12 of 13; 92%).^18^ Domené et al. suggest the investigation for the mutation c.373delT given its prevalence and the simplicity of the technique (enzymatic digestion) to make a definitive diagnosis.^4^ Another study, published in 2011, described a girl with isolated central hypothyroidism with the same mutation found in our patient and proposed a systematic diagnostic workup for CCH to reduce the diagnostic delay in patients with this condition.^13^

As described in the literature, newborn screening test shows high levels of TSH in primary congenital hypothyroidism, whereas normal concentrations of TSH and decreased concentrations of free T4 delay the diagnosis in newborns with CCH. The patient described in the present report was diagnosed at 5 months of age, after exhibiting clinical manifestations of myxedema, although newborn screening had included total T4 that was at a lower limit of the reference standards. The delayed diagnosis resulted in impaired neuromotor and cognitive development. A recent study^22^ showed that neurodevelopmental retardation, following delayed diagnosis and treatment, was present in three cases of CCH (P1a, P2 and P3). In contrast, the younger sibling in kindred 1 (P1b) developed normally following genetic diagnosis and treatment from birth.^22^ These authors and our data show that delayed diagnosis and treatment of severe central hypothyroidism in such cases result in neurodevelopmental retardation. Inclusion of thyroxine (T4) plus thyroxine-binding globulin (TBG), or free thyroxine (FT4) in congenital hypothyroidism (CH) screening, together with genetic case ascertainment enabling earlier therapeutic intervention, could prevent such sequelae.^22^

A recent review summarized the insights into the structural and molecular consequences of the TSHB mutation p.C125Vfs*10, which is associated with isolated TSH deficiency.^23^ Although the mutant Cys125Val appears to negatively influence TSH structure and biological activity, some questions still need to be clarified.^23^

In summary, our case and other ones reported in the literature support the theory that this mutation may be a common cause of isolated TSH deficiency. Isolated TSH deficiency is not detected by routine TSH-based neonatal screening, representing a clinical challenge. Therefore, when possible, molecular genetic study is indicated. Identification of affected and carriers allows the diagnosis, treatment and adequate genetic counseling.
